# The Temporal Pattern of a Lesion Modulates the Functional Network Topology of Remote Brain Regions

**DOI:** 10.1155/2017/3530723

**Published:** 2017-08-03

**Authors:** W. De Baene, G. J. M. Rutten, M. M. Sitskoorn

**Affiliations:** ^1^Department of Cognitive Neuropsychology, Tilburg University, Tilburg, Netherlands; ^2^Department of Neurosurgery, Clinical Imaging Tilburg, Elisabeth-TweeSteden Hospital, Tilburg, Netherlands

## Abstract

Focal brain lesions can alter the morphology and function of remote brain areas. When the damage is inflicted more slowly, the functional compensation by and structural reshaping of these areas seem to be more effective. It remains unclear, however, whether the momentum of lesion development also modulates the functional network topology of the remote brain areas. In this study, we compared resting-state functional connectivity data of patients with a slowly growing low-grade glioma (LGG) with that of patients with a faster-growing high-grade glioma (HGG). Using graph theory, we examined whether the tumour growth velocity modulated the functional network topology of remote areas, more specifically of the hemisphere contralateral to the lesion. We observed that the contralesional network topology characteristics differed between patient groups. Based only on the connectivity of the hemisphere contralateral to the lesion, patients could be classified in the correct tumour-grade group with 70% accuracy. Additionally, LGG patients showed smaller contralesional intramodular connectivity, smaller contralesional ratio between intra- and intermodular connectivity, and larger contralesional intermodular connectivity than HGG patients. These results suggest that, in the hemisphere contralateral to the lesion, there is a lower capacity for local, specialized information processing coupled to a higher capacity for distributed information processing in LGG patients. These results underline the utility of a network perspective in evaluating effects of focal brain injury.

## 1. Introduction

Focal brain lesions (caused by, e.g., stroke or brain tumour) can alter the morphology and function of brain regions remote from the area of structural damage [1, 2]. Several studies have shown prominent functional changes, in patients compared to healthy subjects, in regions distant to the site of damage in situations where the damaged area is normally recruited [3–5]. These remote effects do not conform to a localizationist view but do fit a network perspective that focuses on connectivity and neural communication across regions. According to this network perspective, the effects of focal brain injury should be assessed over entire brain networks instead of just locally at the site of the structural damage [6–9].

Evidence for remote changes after focal damage has been found in different patient populations both at the level of the strength of functional connectivity (e.g., [7, 10–14]) and at the global brain organization level (e.g., [15–17]). Importantly, in several studies, changes in functional connectivity and changes in network organization after focal damage were not only found in the ipsilateral hemisphere but also within the hemisphere contralateral to the lesion (e.g., [18–21]). This is in line with several modelling studies that showed that a virtual lesion can result in changes in functional connectivity and network topology, even of contralesional brain areas (e.g., [8, 22, 23]).

There is significant clinical and experimental evidence that the time-course/kinetics of a lesion influences the functional outcome (e.g., [24–26]). Slowly growing brain lesions generally result in less severe impairments than lesions with an acute onset [27]. Functional compensation and structural reshaping therefore seem to be more effective when brain damage is inflicted over longer periods of time. Several studies have supported this link between the neuroplastic potential following focal brain injury and the temporal pattern of the acquired damage (or “lesion momentum”) (e.g., [28–31]). It remains unclear, however, whether and how the growth rate of the lesion modulates the functional network topology of the remaining healthy areas. An optimal lens to study this is provided by patients with glioma. Gliomas are the most common type of primary brain tumours that are classified based on their malignancy. Low-grade gliomas (LGG, WHO grades I-II) tend to grow more slowly and less aggressively with lower degrees of cell infiltration and proliferation than high-grade gliomas (HGG, WHO grades III-IV). It is estimated that, on average, LGG go undetected for more than a decade before becoming clinically manifested (usually with a seizure; [32]). In contrast, HGG and in particular grade IV glioblastomas grow much faster. Studies suggest a 10-fold difference in growth velocity: about 4 mm/year for LGG compared to about 3 mm/month for HGG [33]. This growth velocity difference could lead to more extensive plastic effects before diagnosis [29, 34] and, therefore, more distinct reorganization of the functional networks in remote areas in LGG compared to HGG patients.

Only few studies have compared the network characteristics in LGG and HGG patients. van Dellen et al. [31] showed functional network differences when comparing LGG patients with HGG patients and healthy controls. No network topology differences were observed between HGG patients and healthy controls. In specific networks (e.g., default mode network and motor network), however, functional connectivity was more disrupted in HGG compared to LGG patients (e.g., [35, 36]). These previous studies examined functional connectivity and functional network topology for a specific network only or at the whole-brain level without differentiating between damaged and undamaged areas. However, insight into the functional organization of the undamaged areas is vital since the extend of functional recovery may be determined by the proportion of the preserved functional network [37]. Furthermore, the severity of behavioural impairment following focal neural damage correlates with the extent of connectivity changes in remote regions [38].

Therefore, in the current study, we examined whether the functional global organization (defined by resting-state connectivity, rs-fMRI) of the undamaged regions differs between LGG and HGG patients. Additionally, we investigated whether specific network topology features of the undamaged areas characterize these two different patient groups. Since the tumour location varied widely across our heterogeneous population of glioma patients, only the hemisphere contralateral to the lesion could be reliably regarded to be tumour-free. Consequently, all analyses were targeted on this hemisphere. In a first step, we used multivariate pattern classification (“machine learning”) to predict the tumour grade (LGG or HGG) at the individual level on the basis of the functional connectivity patterns of the hemisphere contralateral to the lesion. This is based on the presumption that better-than-random classification accuracy can only be obtained if there are indeed differences in the functional connectivity of the hemisphere contralateral to the lesion between the LGG and HGG patients [39]. This is exactly what we found in this study. Therefore, in a second step, we applied graph theoretical analyses [40] to further characterise the network topology of the hemisphere contralateral to the lesion in these two patient groups.

## 2. Methods and Procedure

### 2.1. Study Population

We conducted a retrospective study on the resting-state data of patients recruited from the Elisabeth-TweeSteden Hospital (Tilburg, the Netherlands) from July 2010 to June 2016. Rs-fMRI data was collected as part of a standard presurgical protocol.

Only patients that were eligible for resective tumour surgery for a unilateral left-hemispheric LGG (grade I or grade II) or HGG (grade III or grade IV) (as demonstrated by neuropathological examination) were included in this study. Patients who had undergone a previous tumour resection were excluded from the analyses.

As indicated by the local medical ethics committee, data usage was exempted from approval by an independent ethical committee, since the data were clinically acquired and anonymously processed.

### 2.2. MRI Acquisition Procedure

Images were collected with a 3 Tesla Philips Achieva Scanner (Philips Medical Systems, Best, The Netherlands) using a standard 32-channel radio-frequency head coil. Whole-brain resting-state fMRI data were acquired with a 3D-PRESTO pulse sequence with parallel imaging (TR/TE = 19/27 ms, slice orientation = sagittal, flip angle = 10 degrees, dynamic scan time = 1500 ms, voxel size = 4 × 4 × 4 mm, FOV = 160 × 256 × 256, reconstruction matrix = 40 × 64 × 64, number of volumes = 301). High-resolution whole-brain structural scans were acquired for all patients as reference for the resting-state maps (3D T1-weighted sequence: TR/TE = 8.40/3.80 ms, flip angle = 8 degrees, slice orientation = sagittal, 1 × 1 × 1 mm voxel size, with varying FOV (158 × 254 × 254 in 48 patients; 175 × 240 × 240 in 27 patients; 175 × 288 × 288 in 4 patients, and 160 × 240 × 240 in 1 patient)). All subjects were instructed to relax, but not to sleep, in the scanner while thinking of nothing in particular.

### 2.3. MRI Preprocessing

Scan data was analysed using SPM12 (Wellcome Trust Center for Neuroimaging, London, UK). Preprocessing included realignment, segmentation of the structural image, spatial normalization of the structural and functional images to the template MNI brain, resampling to 2 × 2 × 2 mm cubic voxels, functional outlier detection (based on scrubbing of motion-affected functional volumes), and smoothing using an 8 mm full width at half maximum (FWHM) Gaussian Kernel.

### 2.4. Functional Connectivity

To assess the functional connectivity in each patient, preprocessed rs-fMRI data were first parcellated into 90 regions (45 regions for each hemisphere) of interest (ROIs) using the automated anatomical labelling (AAL) atlas. The representative time series for each ROI were obtained by averaging the BOLD time series over the extent of the parcel. Possible sources of spurious variance were regressed out from the data, including (a) the realignment and scrubbing parameters, (b) the white matter signal, (c) the ventricular system signal, and (d) the whole-brain signal. Finally, linear detrending and temporal band-pass filtering (0.009 to 0.8 Hz) were applied to reduce the influences of low-frequency drift and high-frequency physiological noise.

ROI-to-ROI connectivity maps for the hemisphere contralateral to the lesion were generated using the CONN toolbox [41]. For each subject, this 45 × 45 correlation matrix was created by computing the correlation coefficient between each pair of 45 ROIs of the hemisphere contralateral to the lesion, which were then Fisher transformed.

For the multivariate pattern classification, we removed all 45 diagonal elements and extracted the upper triangle elements of the connectivity maps as classification features. The remaining 990 elements (45 × (45 − 1)/2 = 990) of the connectivity maps served as the feature space for the multivariate pattern classification.

More details on the two different methods that we have used follow below. We will first elaborate on the multivariate pattern classification approach (A) that is used to examine whether the functional global organization of the hemisphere contralateral to the lesion differs between LGG and HGG patients. Secondly, we will portray the graph theoretical analyses (B) that are needed to describe the specific network topology features that characterize our two different patient groups.

#### 2.4.1. Multivariate Pattern Classification

To automatically detect the tumour grade at the individual level on the basis of the contralesional connectivity map, a data-driven method was adopted. It incorporated three steps: feature selection, pattern classification, and permutation testing.

*(1) Feature Selection*. Feature selection can remove noisy or uninformative features before classification. Reducing the number of features does not only speed up computation but can also improve the final classification performance [42, 43]. To this end, we first selected a small set of features with the greatest discriminative power [44]. The discriminative power of a feature can be quantitatively measured by its relevance to classification [45]. In this study, we used the Kendall's tau rank correlation coefficient, which provides a distribution-free test of independence between two variables to measure the relevance of each feature to classification. The discriminative power was defined as the absolute value of the Kendall tau correlation coefficient (see, e.g., [46–48] for a similar approach). We subsequently ranked features according to their discriminative powers and selected the 200 highest ranked features per cross-validation fold (note that similar analyses with the 50, 100, 150, or 250 highest ranked features showed very similar results). Since we used a leave-one-out cross-validation strategy to estimate the generalization ability of the classifiers (see below) and feature ranking is based on a slightly different training data set in each iteration of the cross-validation, the final feature set differed slightly from iteration to iteration. However, out of the 200 final features, 139 consensus features appeared in the final feature set of each cross-validation fold [44]. These consensus features were selected for the subsequent classification analyses.

*(2) Pattern Classification*. Linear kernel support vector machine (SVM) classifiers were used to solve the binary classification problem. SVM algorithms allow classifying individuals based on an underlying multivariate statistical analysis of the data. Based on the training data, the SVM classifier searches for a hyperplane in a high-dimensional space that optimally distinguishes between categories and assigns new, previously unseen data (test data) into the categories based on that optimal hyperplane [49]. To test the ability of the classifier to reliably distinguish between LGG and HGG patients, leave-one-out cross-validation was performed, which gives the most unbiased estimate of the test error [50]. During this cross-validation, each sample is designated the test sample in turns while remaining samples are used to train the SVM classifier. Note that a ten-fold cross-validation, in which the samples are divided into ten sets and in which each set is designated the test set in turns while the remaining sets are used to train the SVM classifier, showed very similar classification performance.

Specificity was defined as the classification accuracy for LGG patients whereas sensitivity was defined as the classification accuracy for HGG patients. The overall classification accuracy was the mean value of specificity and sensitivity.

*(3) Permutation Testing*. We determined the statistical significance of the overall classification accuracy by permutation testing [51, 52]. This involved constructing the null distribution of the classification accuracy by performing 10,000 random permutations of the training category labels and running the classification process including leave-one-out cross-validation on each of these iterations. The *p* value was derived from the number of permutations achieving higher classification accuracy than when the true category labels were used.

#### 2.4.2. Describing Network Topology: Graph-Theoretic Analyses

To investigate the properties of the brain functional networks, each individual's correlation matrix was thresholded into a weighted, undirected graph (i.e., network). Graphs are defined as a set of nodes (ROIs from the correlation matrix which represent brain regions) connected by a number of edges (correlation values above a threshold which represent undirected connections). Given the controversies in the treatment of negative correlations in resting-state studies [53–55], we followed the traditional approach and ignored all negative correlations (e.g., [56–58]). Since the choice of the threshold has a critical effect on the number of edges of the resulting brain networks and thereby influences the topological properties, we estimated and integrated the graph metrics of the brain functional networks over a wide sparsity (defined as the fraction of total possible edges that is present in the graph) ranging from 10% to 50% (in steps of 5%). By using a sparsity-specific instead of a correlation value threshold, we equated the number of edges or wiring cost across subjects [59]. The chosen range is widely accepted since it maintains highly interconnected graphs while they still separate from random topology [60, 61].

**Figure 1:**
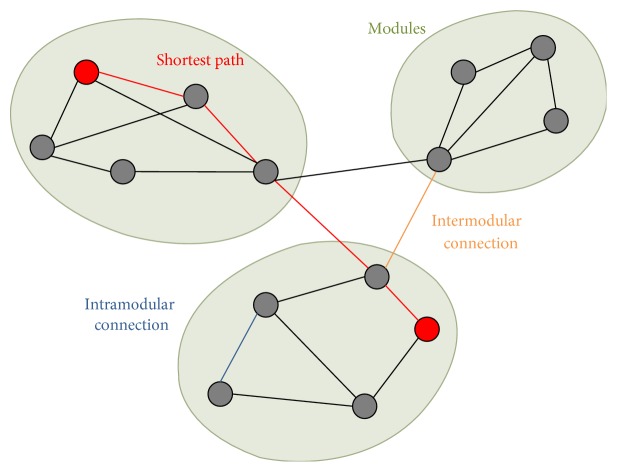
The panel shows an example of a graph, which is a mathematical description of a network, consisting of a collection of nodes and edges. The dots represent nodes, and the lines represent edges connecting the nodes. There are three modules in the graph in which connections within modules (intramodular connections) are much denser than the connections between modules (intermodular connections). The shortest path length describes the minimum number of connections that should be passed to travel between two nodes and is inversely related to the global efficiency.

The network metrics in this study were selected based on their ability to quantify global network characteristics and were computed with the brain connectivity toolbox [58] and are detailed in Figure 1.

*(1) Global Efficiency*. The global efficiency of a network is defined as the average of the inverse of the shortest path length between all nodes (i.e., number of minimum connections that should be passed to join two nodes; [59, 62]). The advantage of global efficiency over the characteristic path length is that only the former can be meaningfully computed on disconnected networks. Global efficiency is thought to represent integration of network-wide communication.

*(2) Local Efficiency*. Contrary to global efficiency, local efficiency is measured on a nodal basis using information about the path length between the neighbours of a single node. It assesses the efficiency of communication between the first neighbours of a node when the node is deleted. High local efficiency indicates that a node is embedded in a richly connected environment. Low local efficiency, by contrast, means that the neighbours of the node are sparsely connected to one another [63]. The local efficiency averaged across all the nodes of a network represents the network's potential for local information transfer [64, 65].

To evaluate the global and local efficiency, these graph metrics have to be normalized because basic low level network properties (such as number of nodes and connection density) influence these measures. Therefore, we benchmarked these metrics to 1000 random reference networks that were randomly rewired to destroy the low level properties but preserved the weight distribution of the entire network [66].

*(3) Modularity*. Modularity quantifies the degree to which a network can be subdivided into separable, nonoverlapping subnetworks or modules in which nodes within the same module are densely interconnected but only have sparse connections with nodes from other modules [67]. The extent of modular organization is assessed by the weighted modularity metric *Q* [68].

*(4) Intramodular Connectivity*. The intramodular connectivity is the sum of all edge weights within a module [69] and reflects the level of local processing within modules.

*(5) Intermodular Connectivity*. The intermodular connectivity is the sum of edge weights between the nodes of different modules [69] and reflects the level of distributed processing between modules.

To avoid differences in these modularity metrics between groups that are merely attributable to global differences in correlation magnitudes across individuals, we divided these modularity metrics by the average connection weights. Additionally, we computed the ratio between the intra- and intermodular connectivity.

Permutation testing [61] was used to determine whether the network properties differed between the LGG and HGG patient groups. First, we calculated the between-group differences for each network metric. To test the null hypothesis that the observed group difference could occur by chance, for each network metric, the group to which each patient belongs was randomly exchanged and the difference between the network metric of the two random groups was computed. This randomization procedure was repeated 10,000 times, resulting in a sampled between-group difference null distribution for each network metric. Finally, for each metric, the observed difference between the LGG and HGG patient groups was assigned with a *p* value by computing the total number of entries from the permutation that exceeded the empirically measured group difference. A significance threshold of *α* = 0.05 was used. The false discovery rate (FDR) correction was applied for multiple comparisons.

Since permutation analyses do not allow for the correction for covariates, we additionally tested group differences in network properties using ANOVAs with age and tumour diameter as covariates. Tumour diameter was defined as the maximum tumour diameter in any dimension, measured digitally on the basis of visually defined signal abnormalities on T1-weighted or FLAIR images.

## 3. Results

### 3.1. Patient Characteristics

From the 84 eligible patients, three subjects were excluded due to conversion problems of the rs-fMRI data. One additional subject was excluded because of the low quality of the rs-fMRI data (temporal signal-to-noise ratio below threshold of 45, which is the lower boundary to reliably detect small (<0.5%) fluctuations, given the number of timepoints used; see [70]). In the analyses, 40 LGG patients (all grade II) and 40 HGG patients (13 grade III; 27 grade IV) were included.

There was no significant difference in gender (Chi square test) or tumour diameter (independent samples *t*-test; *t*(78) = 1.77, *p* > .08) between the LGG and HGG group (Table 1). However, there was a significant difference in age between the two groups (independent samples *t*-test; *t*(78) = 4.66, *p* < .001): LGG patients were significantly younger than HGG patients, as is well-known from the literature (e.g., [71]).

### 3.2. Multivariate Pattern Classification

**Figure 2:**
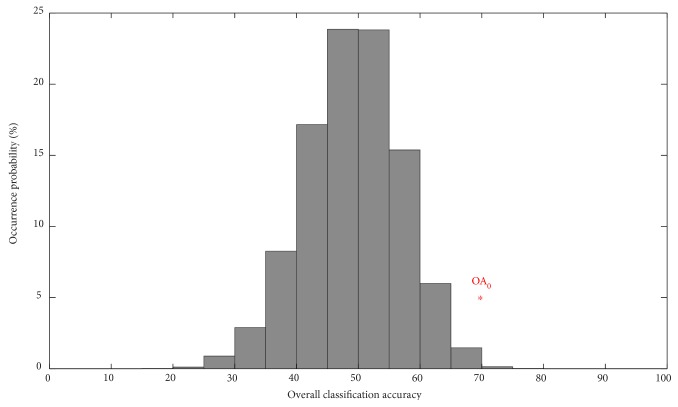
Permutation test results for assessing classifier performance when selecting the 200 most discriminative features. Labels were randomly reshuffled 10,000 times to generate the distribution of the estimate. The red asterisk indicates the overall accuracy obtained by the classifier trained on the real category labels (OA_0_ = 70%).

The classification results indicate that the linear SVM classifier achieved an accuracy of 70% using the 139 consensus features of the hemisphere contralateral to the lesion (70% for LGG patients, 70% for HGG patients). The distribution of the overall classification accuracy for the permuted training data (Figure 2) indicates that the SVM classifier learned the relationship between the data and the group labels with a probability of being wrong lower than .005 (*p* < .005).

### 3.3. Graph-Theoretic Analyses

Both groups showed similar global efficiency and no differences in modularity *Q* (Table 2). In contrast, the intramodular connectivity and intermodular connectivity did differ between LGG and HGG patients: LGG patients showed a larger intermodular connectivity and a smaller intramodular connectivity compared to HGG patients. Furthermore, also the ratio between intra- and intermodular connectivity differed significantly between the two patient groups. Local efficiency was larger in HGG patients than in LGG patients, but this difference did not survive multiple comparison correction.

After correcting for age and tumour diameter differences, the group differences remained significant, except for the intramodular connectivity.

## 4. Discussion

Previous studies have shown that focal lesions can have widespread effects and might lead to functional changes in remote, undamaged areas, even in the hemisphere contralateral to the lesion. These functional changes seem to depend on the temporal pattern of the lesion inflicted to the brain, but up until now, it was unclear whether and how this lesion momentum also modulates the global network organization of the undamaged areas. In the present study, we wanted to examine the possible effects of the growth velocity of a tumour on the functional network topology of the hemisphere contralateral to the lesion, since this was the only brain part that could be reliably regarded to be tumour-free in all our patients. Therefore, we compared the resting-state functional connectivity data of patients with a low-grade and a high-grade glioma, which have a different tumour momentum.

The present results show that there is indeed a difference between patients with a slowly growing low-grade glioma and patients with a faster-growing high-grade glioma in the functional global organization of the hemisphere contralateral to the lesion. First of all, we were able to classify tumour patients with better-than-random accuracy (70%) in the correct tumour grade group (low-grade versus high-grade glioma) only based on the functional connectivity patterns of the hemisphere contralateral to the lesion. Second, LGG patients showed smaller intramodular connectivity, smaller ratio between intra- and intermodular connectivity, and larger intermodular connectivity of the hemisphere contralateral to the lesion than HGG patients. This pattern of results suggests that LGG patients show a lower capacity for local, specialized information processing within modules but a higher capacity for distributed information processing between modules in the hemisphere contralateral to the lesion than HGG patients. The ability for specialized processing within functionally related brain regions arranged in modules is generally referred to as “segregation,” whereas the capacity of the network to rapidly combine and integrate distributed information is referred to as “integration” [72]. The hemisphere contralateral to the lesion of LGG patients is thus characterized by lower segregation and higher integration compared to that of HGG patients. The smaller local efficiency in LGG patients compared to HGG patients, that did not survive multiple comparison correction, is in line with this interpretation.

The current results extent the findings of earlier studies in several ways. A first series of studies compared the whole-brain functional network characteristics of LGG patients and healthy controls, but did not compare LGG and HGG patients as we did here. Across these studies, however, the findings on segregation and integration were inconsistent; whereas initial magnetoencephalography (MEG) studies showed lower segregation and higher integration, particularly in high frequencies, in LGG patients compared to healthy controls [15, 18], a recent fMRI study showed lower functional network integration in LGG patients and no difference between LGG patients and healthy controls on network segregation [17]. A second series of studies did compare LGG and HGG patients, but did not examine the functional global organization of the undamaged areas like we did. van Dellen et al. [31] showed lower functional network integration in combination with lower network segregation in high frequencies and higher network segregation in low frequencies in LGG patients compared to HGG patients. Harris et al. [35] and Mallela et al. [36], by contrast, did not take a whole-brain approach but examined specific networks (the default mode network and the motor network, resp.) and found more disrupted functional connectivity in HGG compared to LGG patients.

One might argue that the observed differences between the LGG and HGG patients in our study are unrelated to their difference in tumour momentum and that they are merely due to the age difference between the two patient groups. This argument could possibly hold for the multivariate pattern classification results. Previous studies (e.g., [73, 74]) have shown that functional connectivity changes with age, thus better-than-random classification accuracy could have been obtained even if the classifier has only captured an age-related difference in functional connectivity between the patients. For the graph-theoretic results, however, we are confident that the differences between LGG and HGG patients cannot be explained by differences in age between the two groups. First of all, the differences between LGG and HGG patients in intermodular connectivity and in the ratio between intra- and intermodular connectivity remained significant after correcting for age differences in the statistical analyses. Second, the pattern of results we observed is in the opposite direction as would be expected based on age differences between the two groups. In our study, the younger LGG patients showed lower segregation compared to the older HGG patients whereas a number of recent studies examining age effects on functional connectivity showed decreased segregation with increasing age (e.g., [75–77]). Furthermore, Geerligs et al. [78] compared healthy populations of young and old participants and reported decreased intramodular connectivity in combination with increased intermodular connectivity with increasing age. Again, the opposite result pattern was found in our study, suggesting that the differences we observed between the two patient groups are not merely caused by age differences between the groups but are, indeed, related to the difference in tumour momentum between LGG and HGG patients.

Although our study provides important new insights on the effects of tumour momentum on the functional global organization of undamaged areas in glioma patients, several questions remain unanswered. The most critical question is whether the differences between the LGG and HGG patients reflect lesion-induced functional abnormalities, compensatory changes, or a combination of both. Because of the absence of longitudinal measures and of a healthy control group, we cannot distinguish between these possibilities in the current study. One possible although highly speculative explanation for the differences between our two patient groups could be that the increased functional integration in LGG patients is due to higher myelination in the hemisphere contralateral to the lesion compared to the HGG patients [44]. This elaboration of the myelin sheath, which increases the efficiency of signal propagation, may be important for efficient information transfer, and, consequently, for the functional integration between areas of different modules [79].

A limitation of the current study is that we have operationalized the tumour momentum of a fairly heterogeneous population of tumours solely based on histological features, as defined in the 2007 CNS WHO classification [80]. However, tumour grade alone may not be the best proxy for tumour momentum. Although low-grade gliomas generally grow much slower compared to high-grade gliomas [33], tumour velocity can also vary within the tumour grade according to the molecular profile [81]. In fact, molecular parameters are increasingly used as predictors for treatment and prognosis [82]. In the update of the CNS WHO classification [83], the WHO introduced, for the first time, a molecular genetic approach for the classification of CNS tumour entities. This integration of phenotypic and genotypic parameters is now common practice in neuro-oncological centers (e.g., [84, 85]). We can assume that the observed effects of tumour momentum on the functional network topology of the hemisphere contralateral to the lesion would even be larger if this momentum would be operationalized on the basis of these combined markers. Unfortunately, to date, the molecular profile is not available for a large part of the included patients. This information will become available for all our patients in the near future.

In the current study, we examined network topology differences between LGG and HGG patients in the hemisphere contralateral to the lesion based on functional connectivity. In future studies, it would also be beneficial to look at differences in the structural network organization between these two groups. The nature of the relationship between the structural and functional network remains a fundamental question [86, 87]. Although, there seems to be no one-to-one correspondence between functional and structural connections and network topologies [88], Meier et al. [89] showed that functional connectivity of the brain can be described by a combination of the underlying structural connections. It remains, however, unclear, whether and how this link might break down due to disease or lesion [90, 91]. To date, only Yu et al. (2016) have examined the structural network changes in brain tumour patients. They observed no differences between a heterogeneous sample of tumour patients and healthy controls on measures of integration and segregation. However, tumour patients showed increased normalized clustering and small worldness, suggesting that the network efficiency of these patients is enhanced compared to the healthy controls.

We have focussed on remote, undamaged areas in the current study because these areas are important for functional recovery [37] and because connectivity changes in these areas seem to determine the severity of behavioural impairment [38]. Several resting-state studies (e.g., [15, 21, 31, 92]) have also reported a similar link between the functional network topology and cognitive functioning. van den Heuvel et al. [93], for instance, showed a strong positive association between the global efficiency of functional networks and intellectual performance in healthy people. Xu et al. [17] showed a similar relationship in low-grade glioma patients: in their study, decreased whole-brain global efficiency was correlated with lower IQ test scores. All studies with brain tumour patients (for a review, see [94]) looked at whole-brain functional networks. Consequently, future studies are needed to examine whether the link between network properties and cognitive functioning only holds at a whole-brain level, is primarily (or exclusively) present for the network topology of the ipsilesional hemisphere, or can also be observed for the hemisphere contralateral to the lesion. These results may reveal potential biomarkers underlying functional recovery.

## 5. Conclusion

In the present study, we examined whether the growth velocity of a tumour modulates the functional network topology of remote brain areas, more specifically of the hemisphere contralateral to the lesion, which plays a crucial role in the functional recovery of brain tumour patients. We therefore compared the resting-state functional connectivity data of patients with a slowly growing low-grade glioma with that of patients with a faster-growing high-grade glioma and observed that the network topology characteristics of the hemisphere contralateral to the lesion differed between these two patient groups. We conclude that the hemisphere contralateral to the lesion of LGG patients is characterized by lower segregation and higher integration compared to that of HGG patients. These results underline the importance of taking a network perspective on the effects of a focal brain injury. Additional research with regard to the underlying mechanisms causing these differences and the possible link between the functional network characteristics of the hemisphere contralateral to the lesion and the cognitive functioning of the patients is warranted.

## Figures and Tables

**Table 1:** 

Characteristics	LGG patients (*n* = 40)	HGG patients (*n* = 40)	*t* or *χ*^2^ value	*p* value
Sex (M/F)	24/16	23/17	*χ* ^2^_(1)_ = .052	.82
Age in years (SD)	38.79 (10.77)	51.28 (13.10)	*t*_(78)_ = 4.66	<.001
Tumour location				
(i) Frontal	14 (+1 BG)	13		
(ii) Temporal	5	12		
(iii) Parietal	1	5		
(iv) Insular	1 (+1 BG)	0		
(v) Occipital	0	1		
(vi) Fronto-parietal	3	1		
(vii) Fronto-insular	7 (+1 BG)	1		
(viii) Fronto-temporo-insular	3 (+1 BG)	2		
(ix) Temporo-insular	2	2		
(x) Temporo-occipital	0	2		
(xi) Parieto-insular	0	1		
Tumour diameter in mm (SD)	55 (19)	48 (16)	*t*_(78)_ = 1.77	.081

Patient characteristics. SD = standard deviation; BG = basal ganglia.

**Table 2:** 

Graph-analytic metric	LGG (*n* = 40) mean (SD)	HGG (*n* = 40) mean (SD)	*p* value (perm)	F-statistic df = (1,79)	*η* ^2^	*p* value (ANOVA)
Global efficiency	.85 (.04)	.85 (.04)	.368	.60	.008	.441
Local efficiency	1.25 (.16)	1.33 (.20)	.044	4.11	.051	.046
Modularity *Q*	.30 (.06)	.32 (.05)	.148	2.59	.033	.112
Intramodular connectivity	1.08 (.03)	1.10 (.03)	.009^∗^	3.48	.044	.066
Intermodular connectivity	.87 (.04)	.85 (.05)	.023^∗^	6.77	.082	.011^∗^
Ratio intra/intermodular connectivity	1.24 (.08)	1.29 (.10)	.008^∗^	6.92	.083	.010^∗^

ANOVAs were corrected for age and tumour. In none of the models, the effect of diameter or age reached significance. SD = standard deviation. As a measure of effect size, eta squared is reported. ∗ = significant after FDR correction.
